# Stage-Specific Non-Coding RNA Expression Patterns during In Vitro Human B Cell Differentiation into Antibody Secreting Plasma Cells

**DOI:** 10.3390/ncrna8010015

**Published:** 2022-02-05

**Authors:** Renee C. Tschumper, Dominique B. Hoelzinger, Denise K. Walters, Jaime I. Davila, Collin A. Osborne, Diane F. Jelinek

**Affiliations:** 1 Department of Immunology, Mayo Clinic, Rochester, MN 55905, USA; tschumper.renee@mayo.edu (R.C.T.); walters.denise@mayo.edu (D.K.W.); 2 Department of Immunology, Mayo Clinic, 13400 East Shea Boulevard, Scottsdale, AZ 85259, USA; hoelzinger.dominique@mayo.edu; 3 Department of Quantitative Health Sciences, Mayo Clinic, Rochester, MN 55905, USA; jaime.davilal@gmail.com (J.I.D.); osborne.andrew@mayo.edu (C.A.O.); 4 Department of Mathematics, Statistics and Computer Science, St Olaf College, Northfield, MN 55057, USA

**Keywords:** non-coding RNA, plasma cells, in vitro B cell differentiation, human B lymphocytes, RNA-seq

## Abstract

The differentiation of B cells into antibody secreting plasma cells (PCs) is governed by a strict regulatory network that results in expression of specific transcriptomes along the activation continuum. In vitro models yielding significant numbers of PCs phenotypically identical to the in vivo state enable investigation of pathways, metabolomes, and non-coding (ncRNAs) not previously identified. The objective of our study was to characterize ncRNA expression during human B cell activation and differentiation. To achieve this, we used an in vitro system and performed RNA-seq on resting and activated B cells and PCs. Characterization of coding gene transcripts, including immunoglobulin (Ig), validated our system and also demonstrated that memory B cells preferentially differentiated into PCs. Importantly, we identified more than 980 ncRNA transcripts that are differentially expressed across the stages of activation and differentiation, some of which are known to target transcription, proliferation, cytoskeletal, autophagy and proteasome pathways. Interestingly, ncRNAs located within Ig loci may be targeting both Ig and non-Ig-related transcripts. ncRNAs associated with B cell malignancies were also identified. Taken together, this system provides a platform to study the role of specific ncRNAs in B cell differentiation and altered expression of those ncRNAs involved in B cell malignancies.

## 1. Introduction

The differentiation of B cells into immunoglobulin (Ig)-secreting plasma cells (PCs) is complex and induced by precise extrinsic signals regulating gene transcription, thus resulting in cell proliferation, expression of biologically relevant cell surface receptors, and induction of a high rate of Ig secretion [1]. To further understand all these processes, isolating and studying B lineage cells, particularly at the PC stage of differentiation, in vivo can be challenging given the low frequency of PCs within the bone marrow, an anatomic site that houses long-lived PCs. To facilitate B cell subtype studies, particularly those focusing on PCs, several groups have devised in vitro PC (IVPC) generating models using multi-step culture systems providing activation molecules and cytokines to mimic the in vivo process [2,3,4,5,6,7,8,9,10]. These studies have demonstrated that it is possible to generate IVPCs and their plasmablast precursors that are phenotypically and molecularly indistinguishable from in vivo cells. In the model described by Jourdan et al. [5,10], IVPCs were shown to acquire expression of key molecules in the differentiation process such as IRF4, AICDA, PAX5, BCL6 and PRDM1 along with increased Ig production. They further identified descriptive gene expression profiles that distinguished starting memory B cells, activated B cells, plasmablasts and PCs, and novel transcriptional networks specific to PC differentiation.

Although gene expression profiles focusing on coding genes provide valuable insight into cell development and malignant transformation, protein-coding genes account for less than 3% of the human genome, with over 70% of the genome being transcribed into non-coding RNAs (ncRNAs) [11], leading to intensive investigations into potential functional roles for ncRNAs. Indeed, both small non-coding micro-RNAs (miRNAs) and long non-coding RNAs (lncRNAs) have been shown to play important roles in many processes including B cell development and malignancy including multiple myeloma (MM), a plasma cell dyscrasia (reviewed in [12,13,14,15,16]). Furthermore, there is a growing recognition of the integral role that ncRNAs play in both Ig gene rearrangement and class switch recombination [17,18,19], underscoring the need to more completely identify patterns of ncRNA expression that occur during B cell activation, proliferation, and differentiation into PCs.

In this study, starting with purified blood B cells, we use an in vitro system largely modeled after published methodologies to focus on the unique aspects of PCs generated in this manner. We present data demonstrating the robust generation of plasmablasts and PCs in our system that are morphologically and phenotypically reflective of the in vivo state. Moreover, RNA-seq analysis of resting B cells, plasmablasts, and PCs allowed us to assess the expression of many protein-coding transcripts involved in B cell differentiation focusing on Ig-related transcripts to characterize the Ig gene repertoire, as well as Ig somatic mutation levels in PCs generated in this manner. RNA-seq analysis also permitted our novel characterization of the expression patterns of ncRNA types across the differentiation continuum in vitro and how they may relate to expression of protein-coding transcripts. Our comprehensive description of PCs generated in this model system supports its utility in discovering potential molecules, particularly ncRNAs, that can disrupt or enhance generation of specific B cell subsets. Furthermore, it has the potential to provide an in vitro system that would permit elucidation of the interplay between coding and non-coding transcripts and accompanying signals underlying acquisition of genetic abnormalities that lead to B cell malignancies.

## 2. Results

### 2.1. Morphology, Expression of Cytoplasmic Ig, and Phenotyping

**Figure 1:**
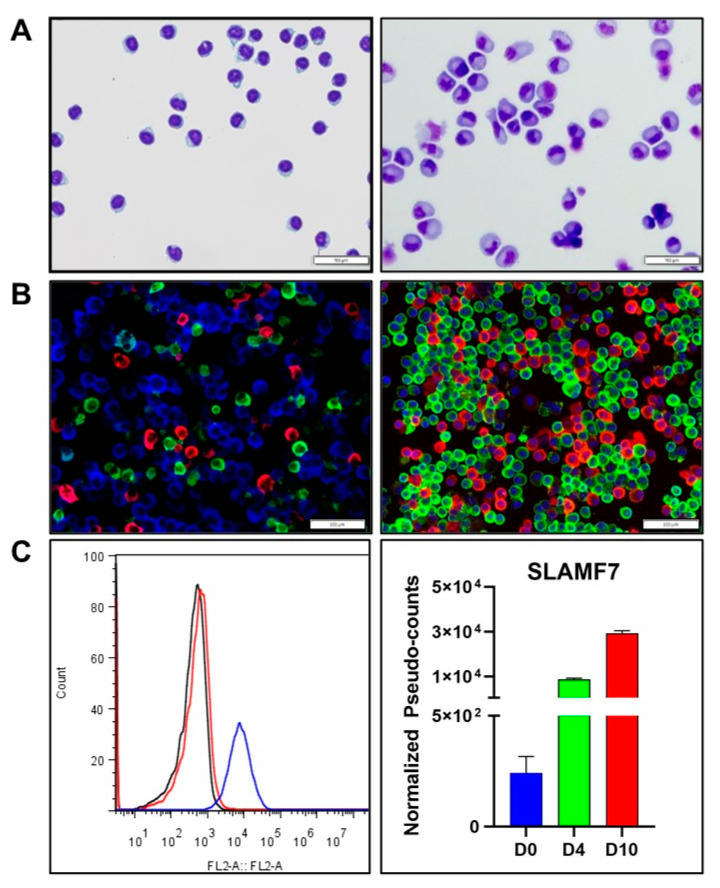
Morphology, cytoplasmic immunoglobulin (cIg) expression and phenotype of B cells differentiated into in vitro plasma cells (IVPC)s. (**A**) Wright staining of day 0 (D0) B cells (left panel) and day 10 (D10) IVPCs (right panel). (**B**) cIg staining of D10 IVPCs. Left panel: D10 IVPCs expressing IgA (red), IgG (blue) and IgM (green). Right panel: D10 IVPCs expressing κ light chain (LC; green), λLC (red), and DAPI (blue). (**C**) SLAMF7 expression. Left panel: D0 cells and D10 IVPCs analyzed by flow cytometry for SLAMF7 expression. Isotype control (black), SLAMF7 D0 B cells (red), SLAMF7 D10 IVPCs (blue). Right panel: SLAMF7 expression by RNA-seq (*p* < 0.0001; D4 = day 4; *n* = 3 at each time point).

Using modifications of previously published protocols [2,3,5,9], IVPCs were generated from normal peripheral blood B cells. By means of a three-stage protocol incorporating various cytokine cocktails, we were able to generate cells that display typical PC morphology and express cytoplasmic Ig (cIg) and PC-specific phenotypic markers. Distinct PC features are present in the day 10 (D10) IVPCs as compared to day 0 (D0) resting B cells, including the expanded cytoplasm, eccentric nucleus and perinuclear hof indicative of protein synthesis and secretion [20] (Figure 1A). Furthermore, D10 IVPCs express cytoplasmic IgA, IgG, and IgM heavy chains and both kappa (κ) and lambda (λ) light chains (LCs; Figure 1B). As expected, the majority of IVPCs expressed IgG and κLCs. SLAMF7 (CD319) has been shown to increase significantly during differentiation of B cells into Ig-secreting PCs [21,22]. SLAMF7 is highly expressed on D10 IVPCs as revealed by flow cytometry (Figure 1C, left panel). Likewise, the RNA-seq data show a highly significant increase in expression of SLAMF7 transcripts across the in vitro activation scheme (Figure 1C, right panel).

### 2.2. Global Expression Patterns during IVPC Differentiation

Using RNA-seq, we investigated transcriptional changes that occur during the in vitro differentiation process from B cells to PCs. Principal component analysis (PCA) shows that B cells isolated from individual donors have consistent phenotypes throughout the differentiation process, with those samples from each time point clustering together (Supplemental Figure S1). D0 and day 4 (D4) plasmablast transcriptomes were more closely related to each other, whereas D10 IVPCs were more divergent. There was more variability between individuals at D10 as compared to D0 and D4, suggesting a high degree of variability in expressed transcripts as cells differentiate, most likely attributable to extensive Ig transcripts and diverse variable, diversity, and joining segment (VDJ) heavy chain recombination.

**Figure 2:**
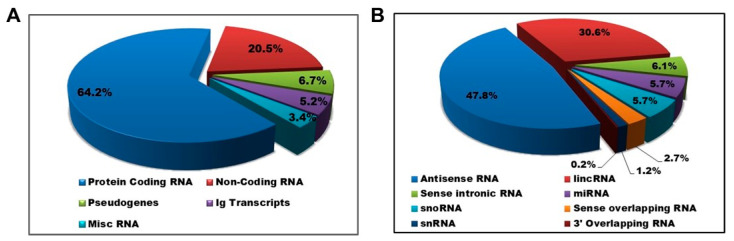
Characterization of differentially regulated RNA transcripts. (**A**) Protein-coding RNAs (excluding immunoglobulin (Ig) transcripts) account for the majority of the differentially expressed RNA transcripts. (**B**) Long non-coding RNAs (lncRNAs) represent 87.4% of the non-coding (ncRNAs) shown in (**A**), with the antisense type representing the majority of lncRNAs.

In evaluating the transcript expression patterns over the course of IVPC differentiation, 4800 transcripts were considered differentially expressed when the log2 fold change (FC) was ≥2 and the false discovery rate (FDR) was ≤0.05 across the 3 time points of D0, D4 and D10 (*n* = 3). Sixty four percent (3091/4800) of the differentially expressed transcripts were non-Ig-related protein-coding transcripts while 20.5% (984/4800) were ncRNA transcripts (Figure 2A). The remaining transcripts were classified as pseudogenes (322/4800; 6.7%) or miscellaneous RNAs (162/4800; 3.4%). Of note, 5.2% of all differentially expressed transcripts were Ig-related transcripts (251/4800). Within the ncRNAs, 87.4% of transcripts were lncRNAs, with antisense RNA being the largest lncRNA class (47.8%; Figure 2B).

Hierarchical clustering of all differentially expressed transcripts across the three time points distinguishes clear patterns of transcript expression during the differentiation process (Supplemental Figure S2). Gene Ontology (GO) analysis of D10 IVPC upregulated transcripts were most relevant to complement activation, immunoglobulin production and immune response (Supplemental Figure S3A), while downregulated transcripts were also involved in the immune response, antigen response and B cell activation (Supplemental Figure S3B) reflecting the transitory expression of transcripts during differentiation and the marked increase in Ig production. Indeed, the expression of representative transcription factors and genes known to be down- and upregulated during the process of B cell differentiation to PCs in vivo, as well as in other models of IVPC generation, is also significantly down- or upregulated in our IVPC model (Supplemental Figure S4; [5,22,23,24,25,26,27,28]). Representative cell cycle transcripts followed the pattern of low expression in D0 resting B cells, increased expression in D4 plasmablasts, and decreased expression in D10 IVPCs in a manner comparable to in vivo B cell differentiation ([29]; Supplemental Figure S5)

### 2.3. Non-Ig Protein-Coding Transcript Expression during IVPC Differentiation

**Figure 3:**
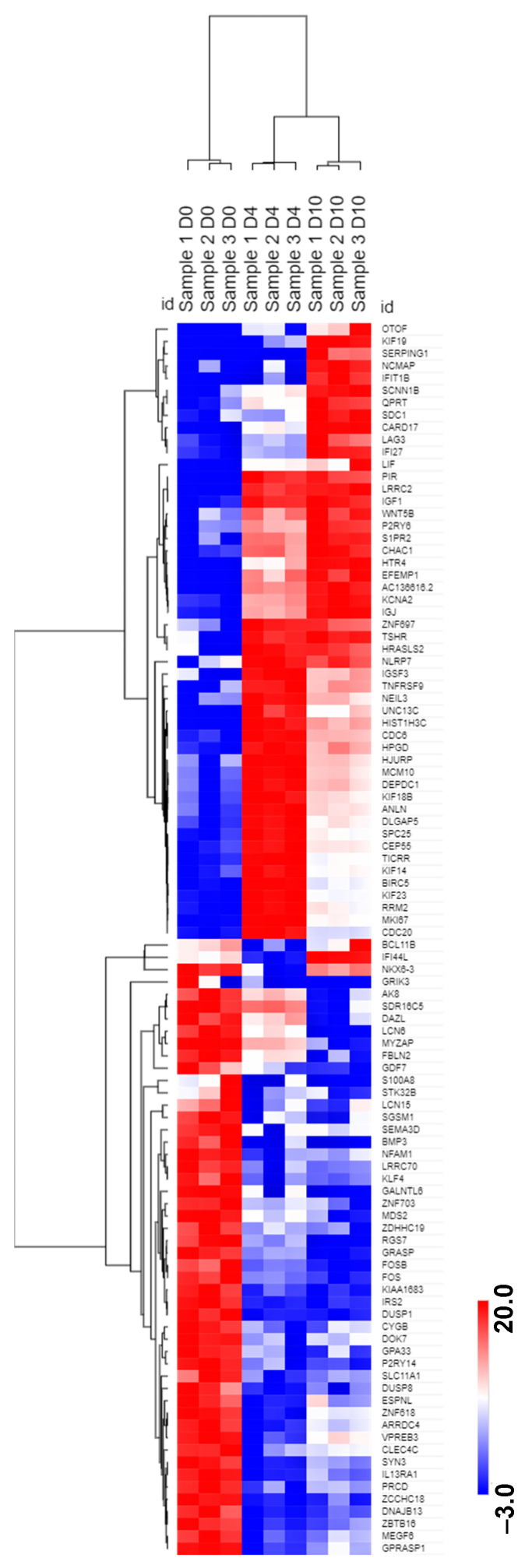
Hierarchical clustering of differentially expressed transcripts during differentiation from D0 resting B cells to D10 IVPCs. The top 100 differentially expressed non-Ig protein-coding transcripts based on fold change (FC) ≥ 2.0 and false discovery rate (FDR) ≤ 0.05 were analyzed by Morpheus software for hierarchical clustering using log2 transformed pseudo-counts and Pearson correlation. The color scale bar represents relative expression based on log2 transformed pseudo-counts.

Focusing on protein-coding transcripts and excluding the abundant Ig-related protein-coding transcripts, the top 100 most significantly differentially expressed transcripts across all three time points again display varied patterns of expression (Figure 3; Supplemental Table S1). Based on FC, the most upregulated transcripts from D0 to D10 IVPCs are HTR4 (FC 10.9) and PIR (FC 10.7). In contrast, BIRC5 (SURVIVIN) and MKI67 are highly expressed in D4 plasmablasts but downregulated in D0 B cells and D10 IVPCs. Of further interest are those transcripts that are downregulated during differentiation from D0 to D10 IVPCs, which includes DUSP1 (FC −7.7), FOS (FC −8.0), and FOSB (FC −6.8).

### 2.4. Ig Protein-Coding Transcript Expression during IVPC Differentiation

When Ig-related transcripts were considered independently, the D10 IVPCs had an overexpression of Ig transcripts including both heavy and light chain transcripts, as would be expected in PCs. With respect to Ig heavy chain (HC) expression, resting B cells (D0) had high levels of IgD and IgM HC transcripts. At D10, IgM transcripts modestly increased (FC = 2.9) and IgD levels decreased significantly (FC = −2.5), in a manner consistent with the normal IgD expression patterns in vivo [30]. IgA and IgG isotype transcripts significantly increased at D10 (FC > 6.0), with the IgG4 subtype having the greatest FC in expression between D0 and D10 IVPCs at 8.7 (Table 1).

Because RNA-seq analysis can also reveal Ig variable (V), diversity (D) and joining (J) segment usage as well as the level of somatic mutations, we next focused on the Ig repertoire of D0, D4, and D10 cells. By aligning to known germline Ig sequences in IMGT/V-Quest (www.imgt.org, last accessed on 18 April 2021) and then assembling the paired ends of D0, D4 and D10 Ig transcripts, we were able to analyze the Ig repertoire. Since the Ig HC variable region (IGHV) is encoded by V, D and J segments, only fragments that could be confidently determined were considered. All but three IGHV transcripts (IGHV3-35, IGHV3-47, and IGHV7-8) and all but two Ig HC diversity region (IGHD) transcripts (IGHD4-4 and IGHD5-5) were found (data not shown). All Ig HC joining region segments (IGHJ) were represented across the differentiation spectrum (data not shown). In D0 cells, the number of unique VDJ combinations ranged from 643 to 863 across all 3 D0 samples and increased to a range of 2524 to 2867 in D10 IVPCs (Table 2). The latter increased numbers most likely reflect increased detection of a wider range of VDJ combinations due to enhanced Ig transcription per cell during differentiation. This is further supported by our observations that when looking at the differential expression of each VDJ combination from D0 to D10, a pairwise t-test for relative frequency showed that there was no significant change greater than 1%, suggesting that the repertoire diversity was not skewed by our stimulation conditions (data not shown).

**Figure 4:**
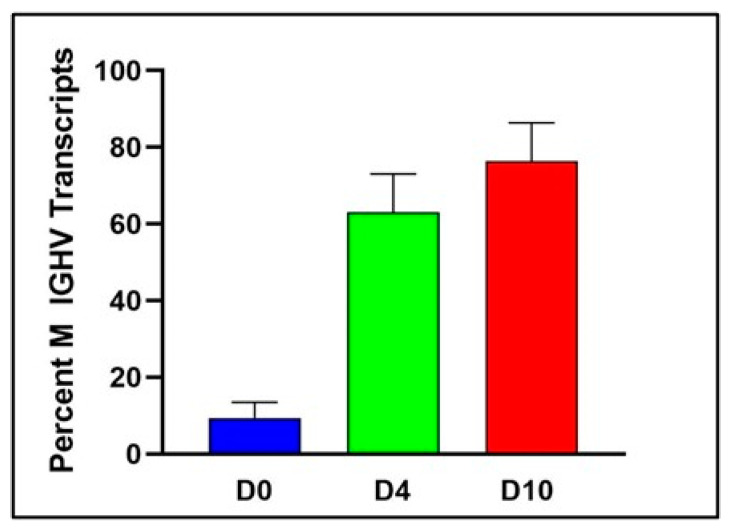
Memory B cells are preferentially expanded in the differentiation process. The percent of unmutated (UM) Ig heavy chain variable region (IGHV) transcripts decreases with differentiation, indicating that cells with a mutated (M) IGHV are preferentially expanded in this in vitro system (*n* = 3; *p* < 0.0001).

Our analysis also permitted an assessment of the extent of somatically mutated (M) IGHV sequences across the differentiation continuum. Indeed, D0 B cells exhibited a high percentage of unmutated (UM) sequences which dropped dramatically in D4 and D10 cells while M sequences increased, suggesting that, in this system, memory B cells are preferentially activated and differentiate into IVPCs (Figure 4).

### 2.5. Non-Protein-Coding Transcript Expression during IVPC Differentiation

**Figure 5:**
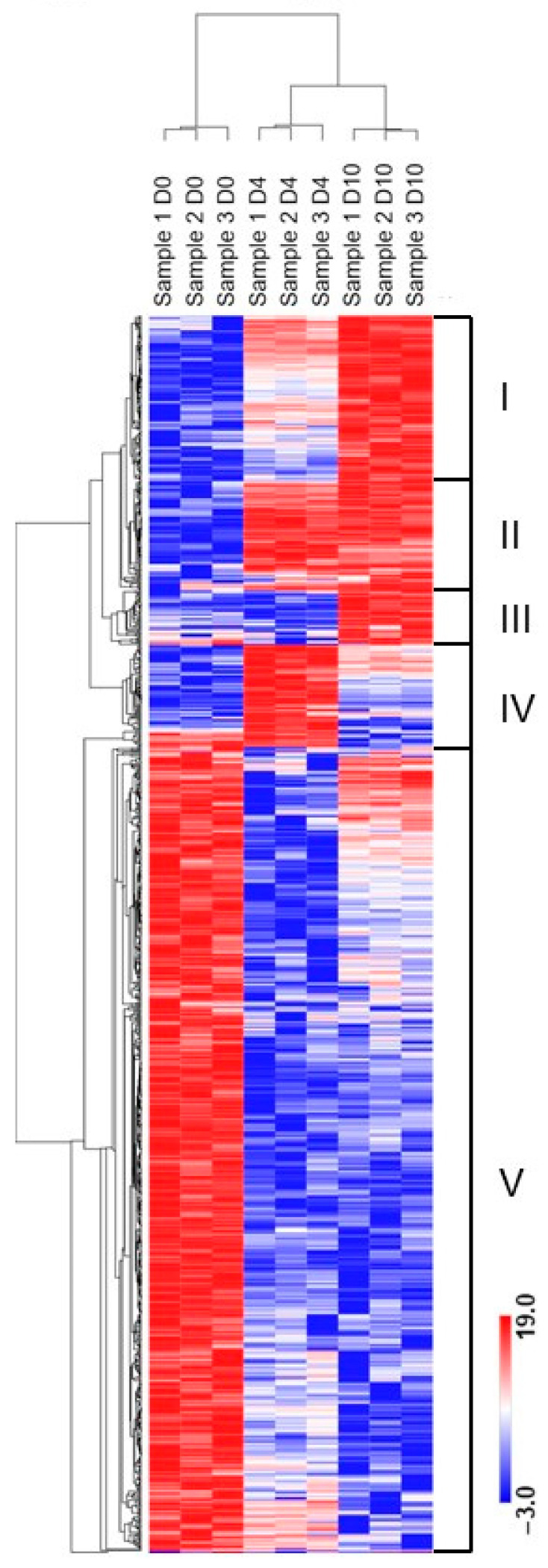
Hierarchical clustering of ncRNAs during the differentiation to IVPCs. A total of 982 ncRNA transcripts were differentially expressed across the transition from D0 resting B cells, D4 plasmablasts and D10 IVPCs based on log2 FC ≥ 2.0 and FDR ≤ 0.05. Five distinctive expression clusters are observed. The color scale bar represents relative expression based on log2 transformed pseudo-counts.

Our RNA-seq data also presented the opportunity to investigate differential expression of all classes of ncRNAs during differentiation of B cells into PCs. Although many ncRNAs still do not have identified functions, an emerging literature demonstrates the importance of ncRNAs in regulating expression of protein-coding genes. Using log2 FC ≥ 2, and FDR ≤ 0.05, 982 non-coding transcripts were considered differentially expressed across all 3 time points and formed 5 distinct clusters of regulation (Figure 5; Supplemental Table S2) representing various classes of ncRNAs (Figure 2B). Although most ncRNAs are expressed at low levels compared to coding genes [31], we found the well-studied long intergenic non-coding RNA (lincRNA) MALAT1 (Cluster V) had the highest expression at each time point across the differentiation continuum. Similarly, the lincRNA MIAT (Cluster I) is also highly expressed across all three time points but slightly elevated in D10 IVPCs (data not shown).

**Figure 6:**
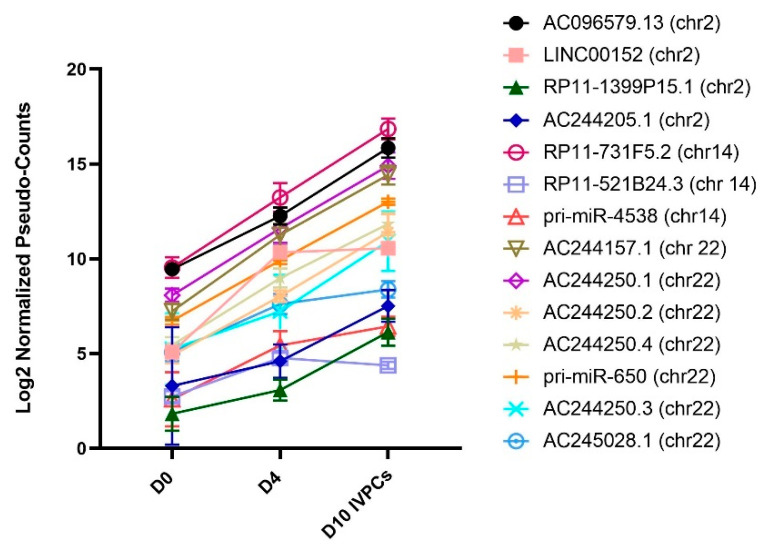
Differentially expressed ncRNA transcripts located in Ig loci. Fourteen differentially expressed ncRNA transcripts are located within Ig loci on chromosomes 2, 14 and 22.

Clusters I–III largely included ncRNAs that were upregulated in D10 IVPCs. Of interest, a number of these are located within the Ig loci on chromosomes 2, 14, and 22. Figure 6 shows the expression levels of ncRNA genes located within the Ig loci, with the majority observed to increase as D0 B cells differentiate into D4 plasmablasts and D10 IVPCs. However, the expression of known or nearby protein-coding targets associated with selected ncRNAs within each of the three Ig loci was observed to be either upregulated or downregulated (Supplemental Figure S6).

**Figure 7:**
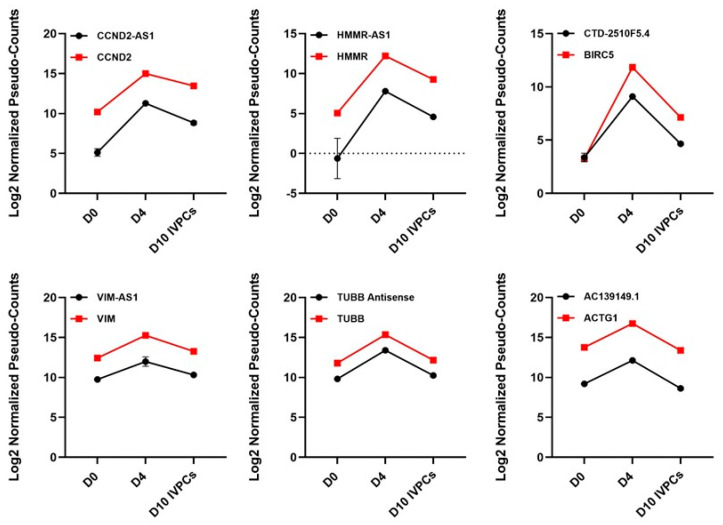
Representative ncRNAs upregulated at D4 (Cluster IV). ncRNAs upregulated at D4 and associated protein-coding transcripts reflect increased proliferation and cytoskeletal conformational changes. Red squares = protein-coding transcripts; black circles = ncRNAs.

Cluster IV comprises ncRNAs that show increased expression at D4 followed by a decrease in D10 IVPCs. This cluster includes ncRNAs associated with proliferation such as CCND2-AS1, HMMR-AS1, and CTD-2510F5.4 and those associated with cytoskeletal proteins such as VIM-AS1, antisense to TUBB, and AC139149.1 reflecting activated, proliferating B cells undergoing cytoskeletal conformational changes (Figure 7).

**Figure 8:**
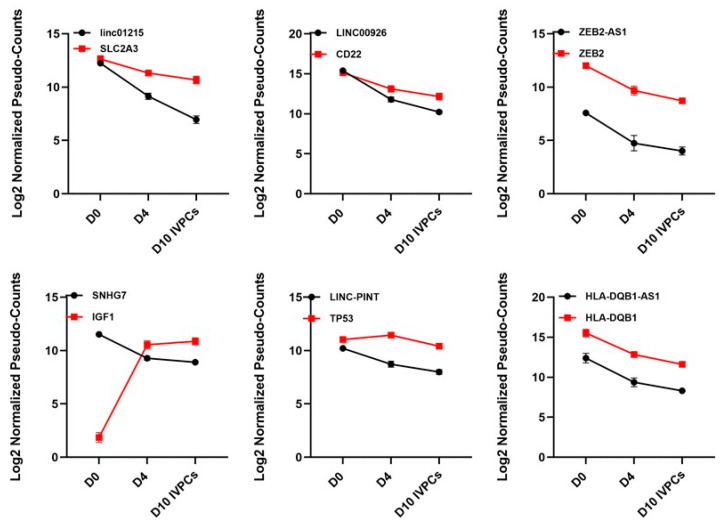
Representative ncRNAs downregulated during B cell differentiation (Cluster V). ncRNAs downregulated during IVPC differentiation and associated protein-coding transcripts. Red squares = protein-coding transcripts; black circles = ncRNAs.

Surprisingly, most differentially expressed ncRNAs fell into Cluster V, with decreasing expression as IVPC differentiation progressed. Thus, it is striking that the expression levels of these ncRNAs were greatly decreased in both D4 plasmablasts as well as D10 IVPCs. Within this group are the ncRNAs LINC01215, LINC00926, ZEB2-AS1, SNHG7, LINC-PINT, and HLA-DQB1-AS1 (Figure 8). It is also notable that MALAT1 is in Cluster V as well. While having the highest expression values across all time points, MALAT1 expression decreased 6.7-fold from D0 to D4 and 1.8-fold from D0 to D10 IVPCs (data not shown).

## 3. Discussion

The generation of Ig-secreting PCs is pivotal to a successful humoral immune response. However, isolating primary human PCs and their precursors for study is technically challenging. Successful models of IVPC generation have proven to be useful tools to better understand B cell differentiation as well as shed new insights into B lineage malignancies [2,4,5,6,8,9,10,24,27,32]. Of note, these studies were focused on characterizing phenotypic changes and identifying protein-coding gene expression profiles, including identification of regulatory transcription factors underlying B cell differentiation. The vast majority of the human genome is transcribed into ncRNAs and there is a growing appreciation for the regulatory role that ncRNAs play in protein-coding gene expression. Differential ncRNA expression in the context of IVPC models is limited. Therefore, in this study, our objective was to utilize RNA-seq and identify a more comprehensive ncRNA expression profile across the differentiation continuum in our modified model system. As the existing literature has demonstrated the impact of culture conditions such as hypoxia, cytokines, and starting B cell phenotype on the resultant IVPCs, it was first necessary for us to characterize and validate the B cell and IVPC phenotype and mRNA expression levels in our in vitro system. As discussed in greater detail below, we analyzed distinctive PC morphological and phenotypic features such as perinuclear hof, cIg and SLAMF7 expression, followed by transcriptional profiling of key transcription factors, cell activation and cell cycling transcripts. Given the robust and hallmark expression of Ig in PCs, we evaluated Ig transcripts to demonstrate that our system generated IVPCs with a diverse Ig repertoire and these were primarily derived from resting memory B cells as revealed by Ig somatic mutation status. Having successfully demonstrated that our system generates PCs that mimic in vivo PCs, we leveraged our model to further identify ncRNA transcripts that are differentially expressed across the B cell differentiation continuum.

While most models described in the literature start with CD27+ memory B cells, we started with total B cells isolated from peripheral blood products and, like Cocco et al. [2], found that memory B cells were preferentially driven to differentiate into PCs in this in vitro system, as evidenced by levels of IGHV region somatic hypermutation. Thus, our work demonstrates that isolation of memory B cells is not a necessary step to generate IVPCs. Because PCs have an inherently high number of Ig transcripts per cell [33], we used a unique method to assess Ig repertoire from our transcriptome data and we were able to verify that the activation conditions did not skew the repertoire. We were also able to assess induction of specific classes of antibody expression and observed decreased IgD expression at D10 with increased expression of IgA and IgG. Our system preferentially activates memory B cells, which typically have undergone isotype switching to IgG or IgA, resulting in loss of expression of IgD. IgD levels also decrease significantly in activated IgM expressing B cells. Thus, our results are entirely consistent with the normal expression patterns of IgD in vivo [30]. Intriguingly, we observed that IgG4 transcripts represented the largest fold increase among Ig isotype transcripts from D0 to D10. Thus, this model system may be useful in specifically studying PC intrinsic gene expression in patients with IgG4-related disease [34] given that IgG4-producing PCs are relatively infrequent and represent 5% or less of all PCs in vivo [35].

In this report, we include a description of differentially expressed protein-coding genes and observed that upregulated transcripts were associated with biological processes involving immunoglobulin production, immune response, positive regulation of B cell activation and B cell receptor signaling while downregulated transcripts again included immune response, but also B cell activation and antigen processing. The primary focus of our study, however, was to gain a better understanding of changes in the transcription of all classes of ncRNAs during the course of primary human B cell activation into proliferating plasmablasts and antibody secreting PCs. Thus, although there are numerous reports describing changes in protein-coding gene expression levels observed in systems generating IVPCs [2,4,5,6,9,10,27], much less is known about the ncRNA transcriptome across the human B cell differentiation continuum. Recent work by Kassambara et al. [10,36], however, did focus on identifying differentially expressed miRNAs and identified numerous stage-specific profiles of B cell development. In the current study, we observed over 900 differentially expressed ncRNA transcripts that demonstrate the existence of a complex network of regulation during differentiation. Hierarchical clustering revealed five distinct patterns of temporally regulated ncRNA transcripts. Indeed, previous studies have identified clear roles for ncRNAs in B cell development and malignancy [15,16,37,38,39,40]. For example, we observed that MALAT1 and MIAT are highly expressed ncRNAs in our system. However, MALAT1 (Cluster V) expression decreases during differentiation but MIAT (Cluster 1) expression increases. Although the current literature on both ncRNAs largely concerns deregulated expression in B cell malignancies [41,42], our results suggest that these ncRNAs, among others, may also be playing important roles, albeit as yet unidentified, in normal human B cell differentiation into PCs.

Our results also revealed the upregulation in expression of several ncRNA genes residing in Ig loci. It remains unclear at this time whether these ncRNAs are highly transcribed because of their location in Ig loci in PCs or are independently expressed and play an important regulatory role in either normal or malignant B cell differentiation or function. In support of the latter concept, LINC00152 on chromosome 2 is highly expressed in an array of tumor types and has been shown to promote metabolism, proliferation, cell survival, growth, and angiogenesis through disruption of various pathways such as PI3K/AKT, mTOR, IL-1, and NOTCH-1 signaling [43]. Our data demonstrate that LINC00152 is highly expressed and protein transcripts described in the literature as being associated with this ncRNA are also differentially regulated during PC differentiation. Another notable example is RP11-731F5.2 (Cluster I), which is located on chromosome 14 within the IGH locus. It is in close proximity to the IGHG2 and IGHG4 genes and has been associated with class switch recombination [44]. However, overexpression of RP11-731F5.2 has also been implicated in other biological systems such as gastric cancer [45] and colorectal cancer [46]. In the context of PCs, transcriptional profiling in MM revealed RP11-731F5.2 to be the most highly expressed lncRNA but still capable of distinguishing hyperdiploid status from non-hyperdiploid in MM [42]. Previously, RP11-7315.2 has been shown to upregulate MS4A1 (CD20) in chronic obstructive pulmonary disease [47]. However, CD20 is known to be downregulated in B cell differentiation and is significantly downregulated in our D10 IVPCs suggesting that this lincRNA may have multiple, diverse functions in B cell differentiation and biological systems. miR-650 on chromosome 22 is located in the Ig λ light chain locus (IgL) and increased expression in B cell chronic lymphocytic leukemia (B-CLL) predicts a more favorable disease course and targets CDK1, ING4 and EBF3 [48]. However, previous studies have shown that miR-650 is transcribed independently of Ig [49]. Indeed, in other tissues, overexpression of mir-650 results in more aggressive cancer such as oral cancer [50] and colorectal cancer [51] by targeting GFI and NDRG2, respectively. In D10 IVPCs, pri-miR-650 is highly expressed and its mature form, miR-650, may be influencing these known targets in a similar manner. Finally, of interest, AC244250.1 (Cluster I) and AC244157.1 (Cluster I) are also located on chromosome 22 and positioned in the IgL locus. These two ncRNAs have yet to be associated with protein-coding transcripts but were highly expressed in our λLC only expressing human MM cell lines (data not shown) suggesting a potential role in MM.

The ncRNAs in Cluster IV are very interesting and may exhibit the most regulatory control in that they are expressly upregulated at D4 (plasmablast state) and then dramatically downregulated upon differentiating into plasma cells. This cluster includes proliferative and cell cycle-related ncRNAs such as CCND2-AS1, HHMR-AS1 [52] and CTD-2510F.4, with the latter playing a role in autophagy [53] and is in close proximity to BIRC5/Survivin. Cytoskeletal coordination is critical in early B cell activation [54] and proliferation [55] and indeed tubulin and actin-related ncRNA transcripts exhibit an increased expression at D4 as do their putative targets of VIM, TUBB and ACTG1 gene transcripts [54,55].

Remarkably, the largest cluster (V) comprises those transcripts that are significantly downregulated upon stimulation (D4) and differentiation (D10). It is tempting to speculate that this cluster includes ncRNAs that antagonize B cell differentiation and that downregulated expression of these ncRNAs is essential to permit expression of protein-coding genes required for B cells to differentiate into antibody secreting PCs. Within Cluster V, we observed that LINC01215 was downregulated as was its associated target, SLC2A3 (GLUT3) [56]. Of interest, LINC01215 was predicted to be highly associated with multiple immune pathways in the context of breast cancer [57]. LINC00926 is linked with CD22, which is a known B cell associated receptor that regulates B cell signaling, proliferation and survival [58]. During differentiation, CD22 expression is lost on PCs and LINC00926 may be regulating that expression. FAM66C decreased expression following activation may be linked to the need for antibody secreting cells to have an active and functioning proteasome pathway to remove excess unfolded Ig molecules. Thus, this ncRNA has been shown to be associated with inhibiting the proteasome pathway in prostate cancer [59]. The lncRNA MATN1-AS1 has been shown to inhibit proliferation in the context of glioblastoma due to its ability to inhibit expression of RELA [60]. Downregulation of this lncRNA in both D4 and D10 B cells may similarly reflect an antagonizing role in B lineage cells as well. Finally, ZEB2 plays a critical role in development of B cells but less is known about its function in mature B cells and PCs [61]. Silencing of ZEB2-AS1 has been shown to downregulate ZEB2 [62], and our data demonstrate that ZEB2 and ZEB2-AS1 are both downregulated in D10 IVPCs.

In conclusion, we demonstrate an effective method for generating IVPCs from total blood B cells that reflect the in vivo state, providing a model for downstream applications and investigations into normal B cell biology and malignancy. Using RNA-seq, we were able to distinguish specific expression profiles across the continuum of B cell differentiation into PCs. Of particular interest was the multitude of ncRNA transcripts that were differentially expressed across the three time points, confirming an underlying and important role for ncRNAs in B cell development and differentiation. As the role of ncRNAs has largely been characterized using primary malignant and normal B cells isolated from humans and mice [36,38,42,63,64,65,66,67], isolation of sufficient numbers of pure cells remains challenging. This in vitro system provides an alternative strategy to circumvent this challenge. Of further note, during the tightly regulated process of B cell differentiation, there are multiple opportunities for errors that may predispose to B cell malignancy. This methodology would facilitate the study of distinct B cell subsets without extensive sample collection. Indeed, inducing malignant transformation during differentiation, possibly by introducing defined genetic elements similar to those experiments performed in mice by Högstrand et al. [68] would further our understanding of B cell malignancies at all stages [69].

## 4. Materials and Methods

### 4.1. Isolation of Peripheral Blood Mononuclear Cells

Peripheral blood mononuclear cells (PBMCs) were isolated from leukoreduction system chambers after plateletpheresis of healthy volunteer blood donors through the Division of Transfusion Medicine at Mayo Clinic, Rochester, MN, USA [70]. B lymphocytes were isolated from PBMCs by negative selection using the Easy Sep Human B Cell Enrichment kit (StemCell Technologies, Cambridge, MA, USA) and a Robosep-S instrument (StemCell Technologies, Cambridge, MA, USA) following the manufacturer’s protocols.

### 4.2. Cell Culture

#### 4.2.1. Plasmablast Generation (Day 0–4)

The B cell in vitro culture and differentiation scheme used in this study was developed using modifications of previously published protocols [2,3,5,9]. All B cell cultures were carried out using Iscove’s modified Dulbecco’s medium (IMDM), with GlutaMax-I (Gibco/Thermo Fisher Scientific, Waltham, MA, USA) with 10% fetal bovine serum (FBS; Atlanta Biologicals, Flowery Branch, GA, USA) and supplemented with 50 µg/mL human transferrin and 5 µg/mL insulin (both from Sigma-Aldrich, St. Louis, MO, USA). Purified B cells were initially cultured for four days in this media with the following cytokines: soluble CD40L (0.1 µg/mL); enhancer for ligands (0.1 µg/mL; both from Enzo Life Sciences, Farmingdale, NY, USA); CpG phosphororthioate oligodeoxynucleotide 2006 ([71]; (10 µg/mL; synthesized by IDT, Coralville, IA, USA); IL-2 (50 ng/mL); IL-10 (50 ng/mL); and IL-15 (10 ng/mL; all from Peprotech, Rocky Hill, NJ, USA). Cells were plated in 6 well plates at a density of 100,000 cells/mL in 5 mL per well.

#### 4.2.2. Plasma Cell Generation (Day 4–10)

At D4 of culture, plasmablasts were collected, washed and re-cultured in IMDM GlutaMax-I plus 10% FBS, transferrin and insulin in 6 well plates at a density of 500,000 cells/mL of media in 5 mL per well with the following cytokines: IL-2 (50 ng/mL); IL-10 (50 ng/mL); IL-15 (10 ng/mL); and IL-6 (50 ng/mL; a kind gift from Novartis, Basel, Switzerland). At day 7 (D7) of culture, cells were collected, washed and re-cultured in IMDM GlutaMax-I plus 10% FBS, transferrin and insulin in 6 well plates at a density of 500,000 cells/mL of media in 5 mL per well with the following cytokines: IL-15 (10 ng/mL); IL-6 (50 ng/mL); and 500 U/mL IFN alpha (R&D Systems, Minneapolis, MN, USA). On D10, cells displayed an aggregate phenotype similar to in vivo PCs and are thereafter referred to as IVPCs.

### 4.3. Immunophenotypic Analysis

At various points along the time course, cells were collected onto glass slides by cytospin centrifugation and after fixation for 10 min in 95% ethanol, slides were incubated for 10 min in permeabilization buffer (PBS plus 0.2% Triton X-100; Sigma-Aldrich, St. Louis, MO, USA), rinsed briefly in PBS, and stained for cytoplasmic Ig for 45 min at 37 °C in a humidified chamber. After washing with permeabilization buffer and air drying, the slides were cover slipped with Vectashield mounting media with or without DAPI (Vector Labs, Burlingame, CA, USA). Various combinations of the following anti-human Ig antibodies were used at a 1:100 dilution: IgA-TRITC; kappa-FITC; IgM-FITC; lambda-TRITC (all from Southern Biotech, Birmingham, AL, USA); and IgG-AMCA (Vector Labs, Burlingame, CA, USA). Freshly isolated B cells (D0) and D10 IVPCs were collected onto glass slides by cytospin centrifugation and stained for standard morphological features using Wright stain (Thermo Fisher Scientific, Waltham, MA, USA). D0 B cells and D10 IVPCs were analyzed by flow cytometry using a monoclonal PE antibody to SLAMF7/CD319 (Biolegend, San Diego, CA, USA) with an appropriate isotype matched control antibody (Biolegend, San Diego, CA, USA).

### 4.4. RNA-Seq and Bioinformatics

At each phase of differentiation, cells were collected, and RNA isolated using the RNeasy Micro Kit (Qiagen, Germantown, MD, USA). RNA was submitted to the Mayo Clinic Medical Genome Facility, Genome Analysis Core (Rochester, MN, USA), where RNA libraries were prepared using 100 ng of high-quality total RNA according to the manufacturer’s instructions for the TruSeq RNA Sample Prep Kit v2 (Illumina, San Diego, CA, USA) employing poly-A mRNA enrichment using oligo dT magnetic beads. The final adapter-modified cDNA fragments were enriched by 12 cycles of PCR using Illumina TruSeq PCR primers. The concentration and size distribution of the completed libraries was determined using Fragment Analyzer (AATI, Ankeny, IA, USA) and Qubit fluorometry (Invitrogen, Carlsbad, CA, USA).

Libraries were sequenced at 39 to 68 million fragment reads per sample following Illumina’s standard protocol using the Illumina cBot and HiSeq 3000/4000 PE Cluster Kit. The flow cells were sequenced as 100 × 2 paired end reads on an Illumina HiSeq 4000 using a HiSeq 3000/4000 sequencing kit and HCS HD 3.4.0.38 collection software. Base-calling was performed using Illumina’s RTA version 2.7.7. The transcriptome data (RNA-seq) generated for this study were deposited in the GEO repository with the accession number GSE194123. Sequencing reads were analyzed using the RNA-seq bioinformatics pipeline MAP-RSeq [72].

Differential expression analysis was conducted using edgeR (ver 3.28.1; [73]) and R (ver 3.6.2; www.R-project.org, last accessed on 18 March 2021) [74], with counts normalized using Trimmed Mean of M values (TMM) [75]. Transcripts were considered to be differentially expressed as defined by log2 FC ≥ 2 and FDR ≤ 0.05. ANOVA-like QL F-test analysis using function glmQLFit from edgeR was performed with emphasis on the differential expression across all three time points. Normalized pseudo-counts, which represent the equivalent counts if the number of reads had all been equal under the fitted model, were generated using exactTest from edgeR. Principal Component Analysis (PCA) was conducted using pseudo-counts.

For IGH expression analysis, the RNA-seq reads were aligned using STAR aligner [76] and reads aligning to the chromosomal coordinates of the IGH locus, as well as their read pair mate, were extracted. Random hexamers were trimmed, and the pairs submitted to IMGT HighV-Quest (www.imgt.org, last accessed on 20 April 2021) [77]. Each pair mate of the read pair was annotated with the appropriate V, D and J segment. If there was sufficient length coverage (minimum of 40 nucleotides for each read), the two read mate annotations were then collapsed into a single VDJ call. The VDJ calls were then filtered to keep those calls in which only one confident gene was called for each V, D, and J locus, as well as setting a minimum threshold of 95% identity specifically for the V gene for each call. The number of V region mutations was determined within each read of the complete VDJ call as compared to the reference gene annotation.

Heat maps were generated by Morpheus matrix visualization and analysis software (https://software.broadinstitute.org/morpheus, last accessed on 3 May 2021) using the log2 normalized pseudocolor values and Pearson correlation. GO analyses were performed using GeneCodis (https://genecodis.genyo.es/, last accessed on 20 June 2021) to generate meaningful annotations of genes and gene products focusing on biological processes, cellular components, and molecular functions.

## Figures and Tables

**Table 1:** Expression of Ig heavy chain (HC) gene transcripts during IVPC differentiation.

IGH Transcript	FC *	D0 **	D10
IGHA1	7.3	3.8 × 10^3^	6.7 × 10^5^
IGHA2	7.1	7.9 × 10^2^	1.2 × 10^5^
IGHD	−2.5	3.3 × 10^4^	1.0 × 10^4^
IGHG1	7.4	2.0 × 10^4^	3.3 × 10^6^
IGHG2	7.6	2.1 × 10^3^	4.1 × 10^5^
IGHG3	6.4	6.5 × 10^3^	5.6 × 10^5^
IGHG4	8.7	3.9 × 10^2^	1.3 × 10^5^
IGHM	2.9	1.3 × 10^5^	9.7 × 10^5^

* FC based on D10 IVPCs vs. D0 resting B cells. ** Expression levels based on normalized pseudo-counts (*n* = 3).

**Table 2:** Unique variable, diversity, and joining segment (VDJ) combinations in resting B cell to IVPC differentiation.

	D0	D4	D10
Sample 1	643	2083	2617
Sample 2	863	2813	2867
Sample 3	724	1415	2524

## Data Availability

The transcriptome (RNA-seq) data generated for this study were deposited in the GEO repository with the accession number GSE194123.

## Supplementary Materials

The following supporting information can be downloaded at: https://www.mdpi.com/article/10.3390/ncrna8010015/s1, Figure S1: Differentiation stage-specific transcript expression; Figure S2: Hierarchical clustering of differentially expressed transcripts during the differentiation from D0 resting B cells to D10 IVPCs; Figure S3: (A) Gene ontology (GO) annotation of upregulated transcripts, (B) GO annotation of downregulated transcripts; Figure S4: Regulation of transcription factors and specific genes in B cell differentiation; Figure S5: Cell cycle transcripts in B cell differentiation; Figure S6: Representative ncRNAs located in Ig loci and their respective known or suggested associated protein-coding genes; Table S1: Top 100 differentially expressed protein-coding transcripts (excluding Ig transcripts) as clustered in Figure 3; Table S2: Differentially expressed ncRNA transcripts during differentiation from B cells to IVPCs
