# Supplementary material for: Stage-Specific Non-Coding RNA Expression Patterns during In Vitro Human B Cell Differentiation into Antibody Secreting Plasma Cells
Source: Noncoding RNA. 2022 Feb 5;8(1):15. doi: 10.3390/ncrna8010015 (PMC8878715; doi:10.3390/ncrna8010015)
Supplement: Supplementary file 1 [file ncrna-08-00015-s001.zip › Supplemental Figures.pdf]

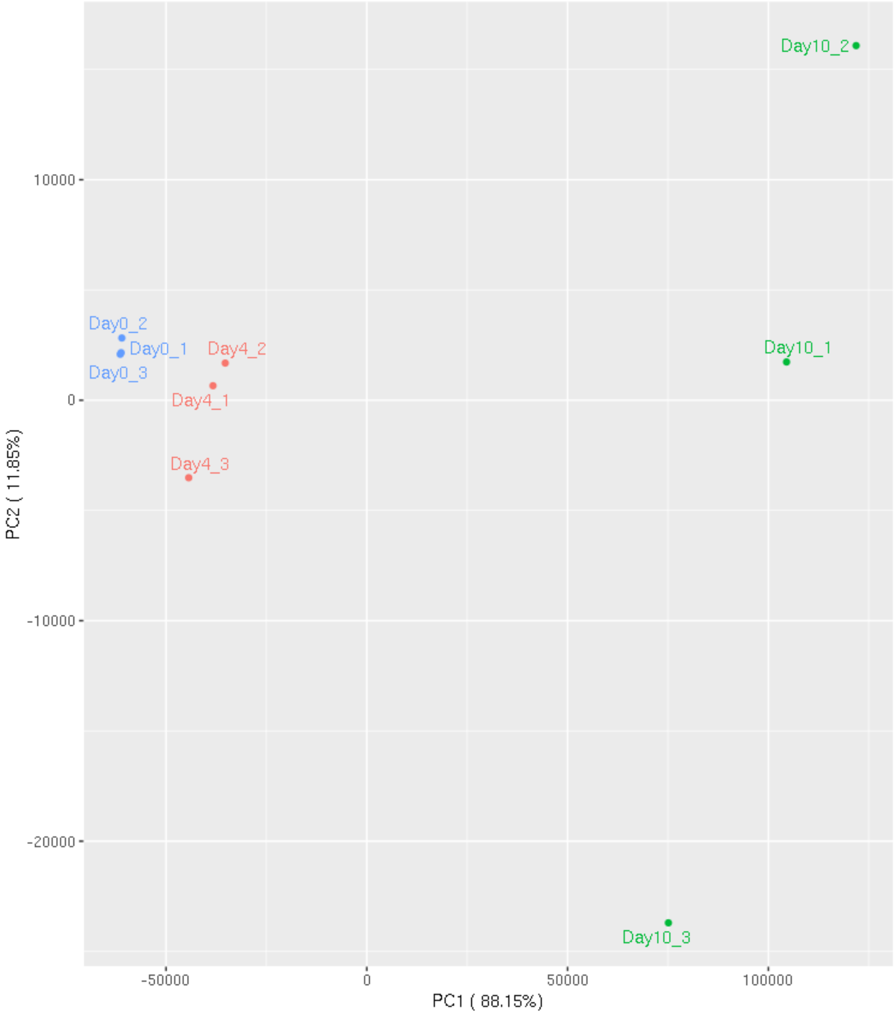

Supplemental Figure S1. Differentiation stage-specific transcript expression. Principal component analysis (PCA) shows that B cells cluster according to their activation state from D0 (blue), D4 (red) and D10 IVPCs (green).

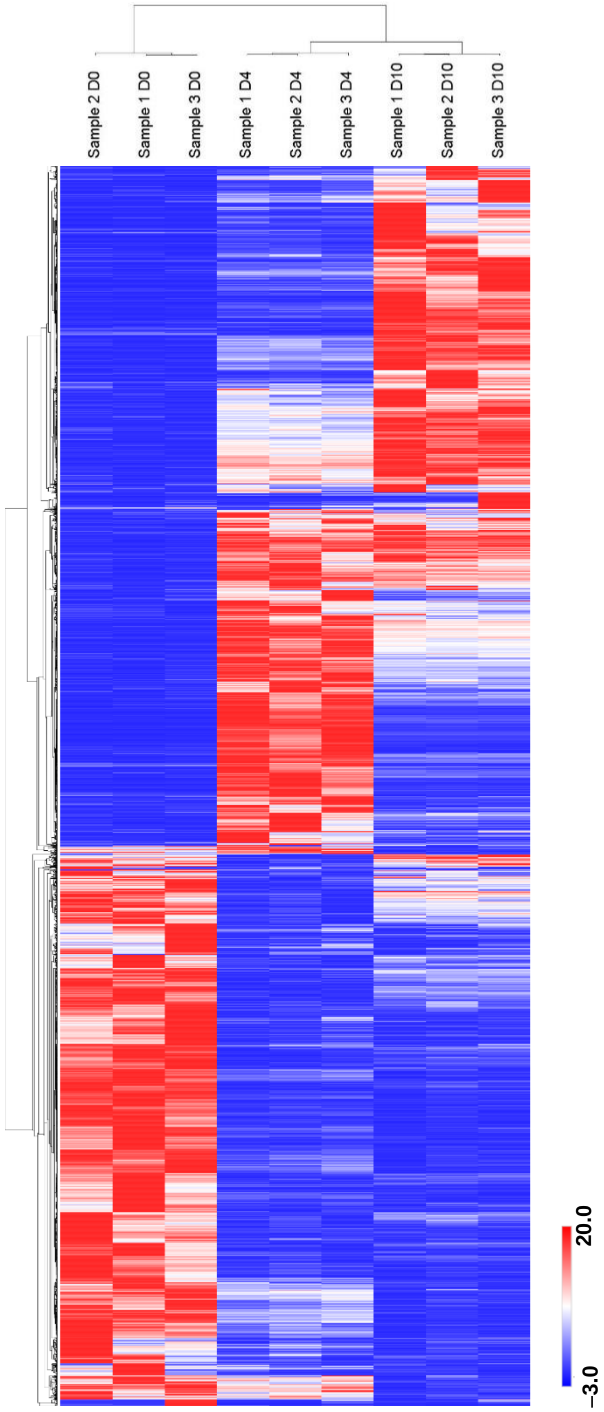

Supplemental Figure S2. Hierarchical clustering of differentially expressed transcripts during the differentiation from D0 resting B cells to D10 IVPCs. Differentially expressed transcripts based on fold change (FC) ≥ 2.0 and false discovery rate (FDR) ≤ 0.05 when including all transcripts. were analyzed by Morpheus software for hierarchical clustering using log2 transformed pseudo-counts and Pearson correlation. The color scale bar represents relative expression based on log2 transformed pseudo-counts.

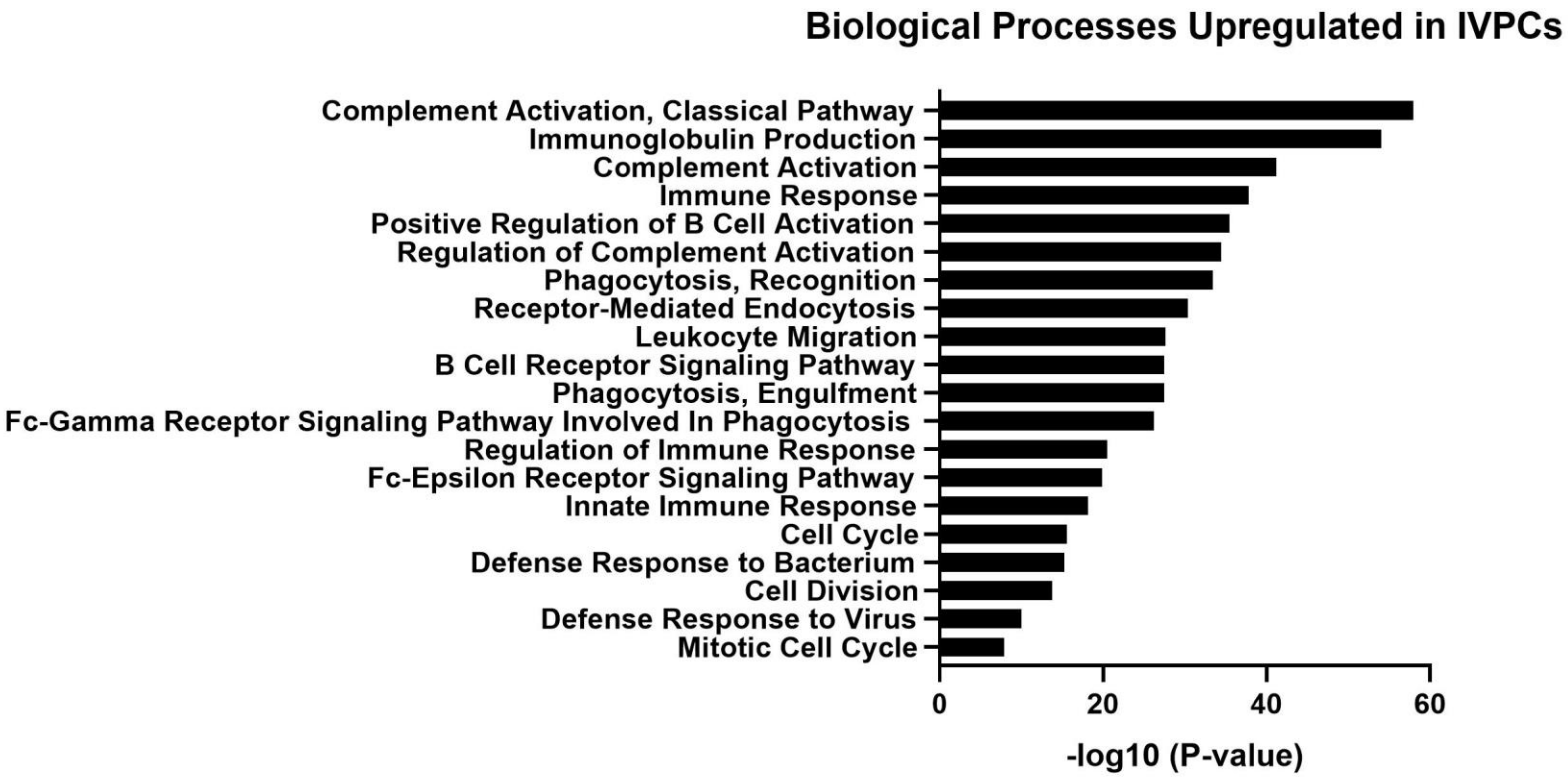

Supplemental Figure S3A. Gene ontology (GO) annotation of upregulated transcripts**.** D10 IVPC upregulated transcripts are involved in complement activation, immunoglobulin production and immune response. The top 20 Biological Processes are shown.

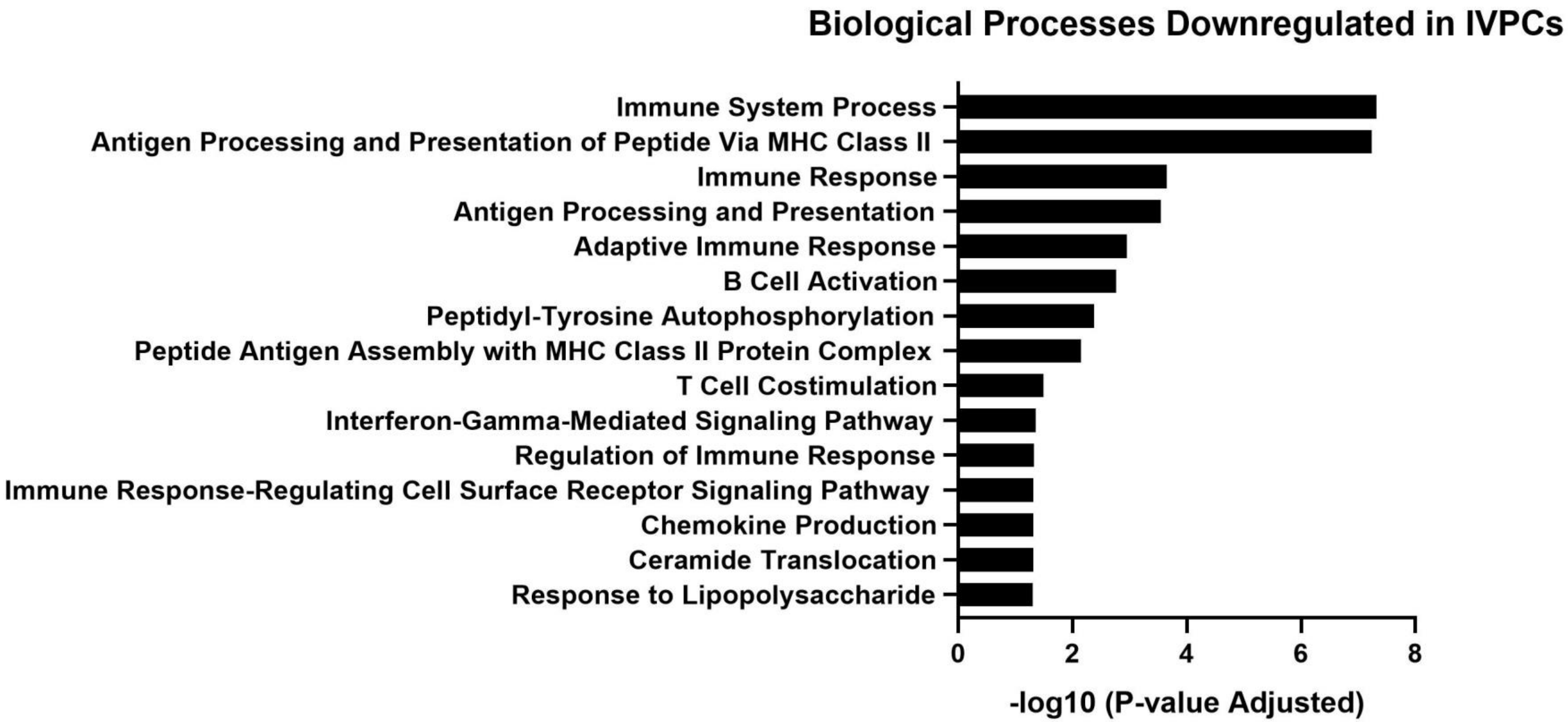

Supplemental Figure S3B. GO annotation of downregulated transcripts. D10 IVPC downregulated transcripts are involved in immune response, antigen response and B cell activation.

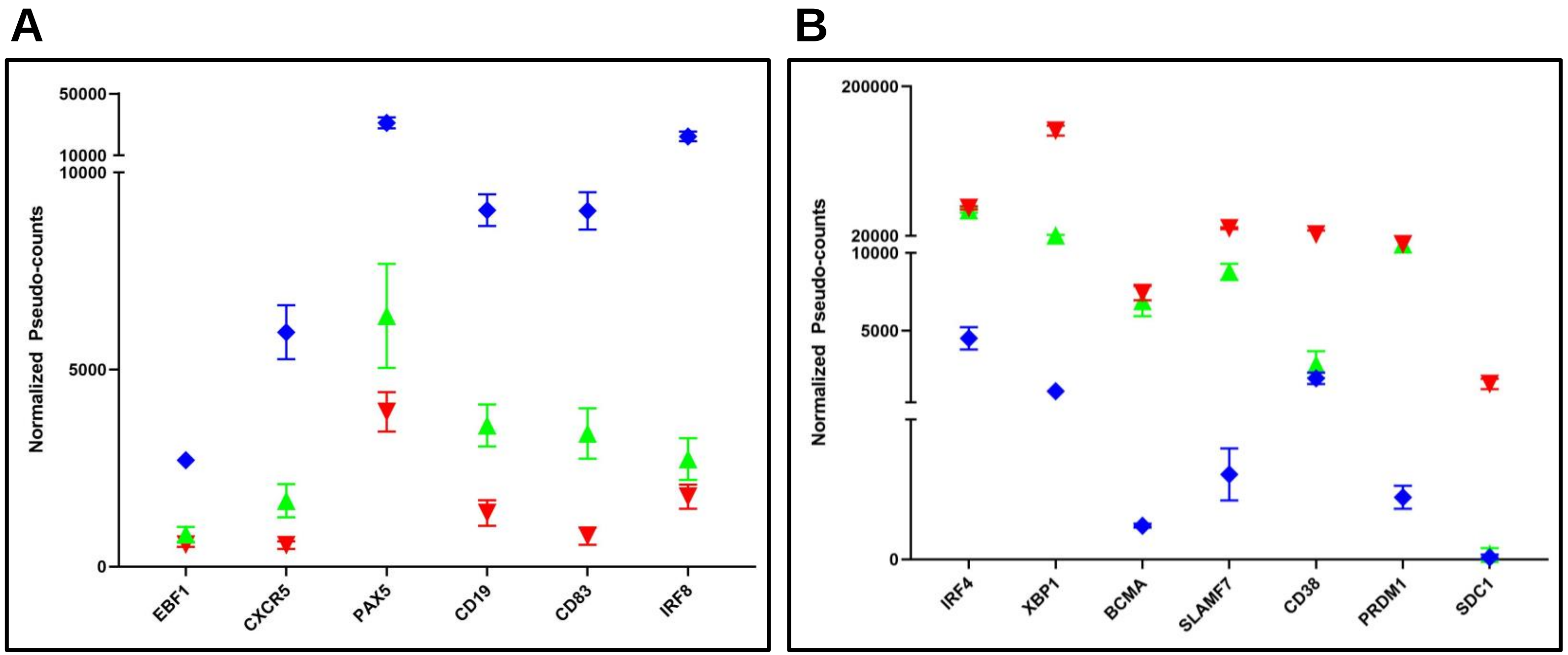

Supplemental Figure S4. Regulation of transcription factors and specific genes in B cell differentiation. Expression of transcription factors and genes known to be **(A)** downregulated; and **(B)** upregulated in B cell differentiation to PCs *in vivo* are also significantly down- or up-regulated in IVPCs ($p<0.0001$). D0 = blue; D4 = green; D10 IVPCs = red (n=3).

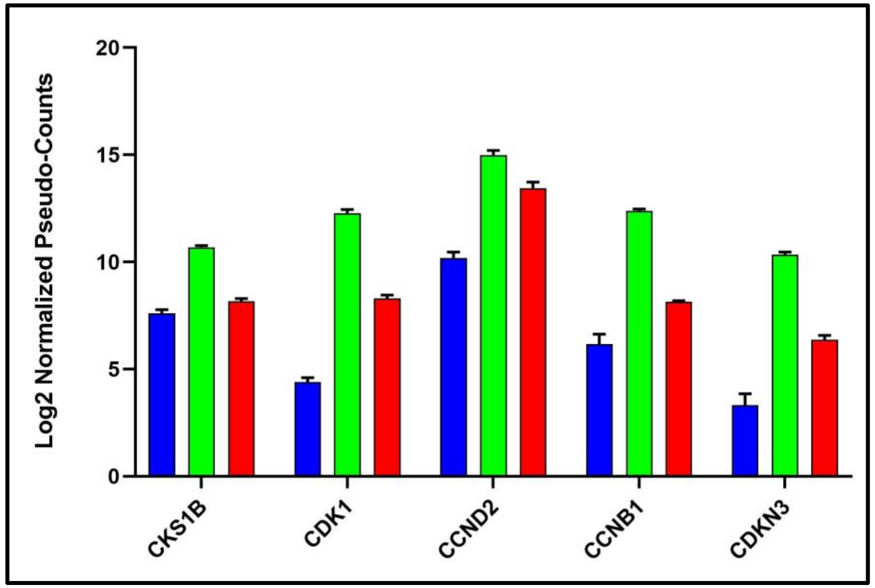

Supplemental Figure S5. Cell cycle transcripts in B cell differentiation. Representative cell cycle transcripts followed the pattern of low expression in D0 B cells, increased expression in D4 plasmablasts and decreased expression in D10 IVPCs. D0 = blue; D4 = green; D10 IVPCs = red (n=3).

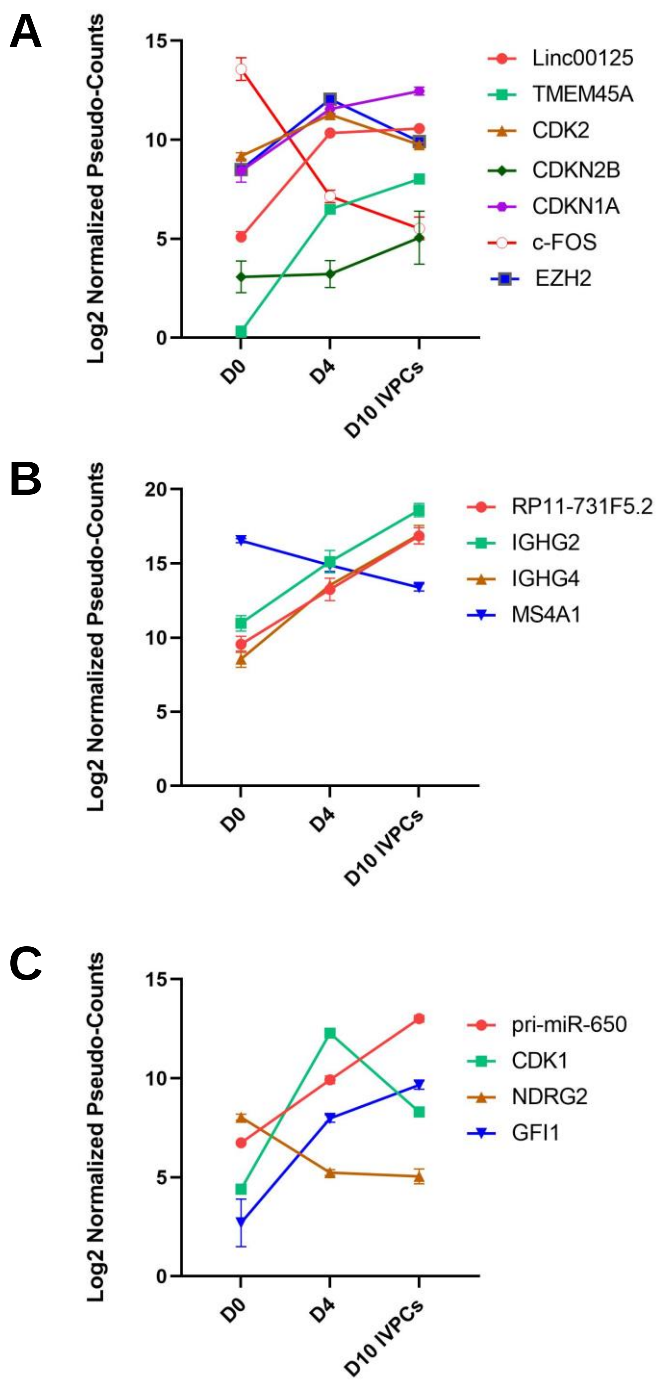

Supplemental Figure S6. Representative ncRNAs located in Ig loci and their respective known or suggested associated protein-coding genes. **(A)** Linc00152 on chr2, **(B)** RP11-731F5.2 on chr14, and **(C)** pri-miR-650 on chr22.
