# Supplementary material for: Stage-Specific Non-Coding RNA Expression Patterns during In Vitro Human B Cell Differentiation into Antibody Secreting Plasma Cells
Source: Noncoding RNA. 2022 Feb 5;8(1):15. doi: 10.3390/ncrna8010015 (PMC8878715; doi:10.3390/ncrna8010015)
Supplement: Supplementary file 1 [file ncrna-08-00015-s001.zip › Supplemental Table S1.pdf]

Supplemental Table S1. Top 100 differentially expressed protein-coding transcripts (excluding Ig transcripts) as clustered in Figure 3.

Upregulated Transcripts	Ensembl ID	FC	p-value*	Downregulated Transcripts	Ensembl ID	FC	p-value
OTOF	ENSG00000115155	**9.0**	4.92E-05	BCL11B	ENSG00000127152	-5.3	5.38E-03
KIF19	ENSG00000196169	**10.0**	8.49E-11	IFI44L	ENSG00000137959	-5.1	2.34E-12
SERPING1	ENSG00000149131	**9.6**	6.60E-09	NKX6-3	ENSG00000165066	-7.3	8.62E-10
NCMAP	ENSG00000184454	**8.9**	2.17E-12	GRIK3	ENSG00000163873	**-7.8**	1.23E-06
IFIT1B	ENSG00000204010	**8.7**	1.27E-08	AK8	ENSG00000165695	**-7.8**	1.22E-09
SCNN1B	ENSG00000168447	**9.2**	2.92E-14	SDR16C5	ENSG00000170786	**-7.0**	3.90E-07
QPRT	ENSG00000103485	**8.3**	8.90E-15	DAZL	ENSG00000092345	**-7.2**	7.17E-09
SDC1	ENSG00000115884	**8.4**	1.80E-08	LCN6	ENSG00000204003	-4.2	2.11E-13
CARD17	ENSG00000255221	**9.9**	1.54E-10	MYZAP	ENSG00000263155	**-9.0**	1.37E-13
LAG3	ENSG00000089692	**8.9**	3.05E-13	FBLN2	ENSG00000163520	**-8.1**	3.72E-13
IFI27	ENSG00000165949	**9.7**	4.76E-21	GDF7	ENSG00000143869	**-6.5**	1.29E-13
LIF	ENSG00000128342	**9.9**	3.97E-02	S100A8	ENSG00000143546	**-7.6**	3.30E-06
PIR	ENSG00000087842	**10.7**	1.68E-11	STK32B	ENSG00000152953	**-7.2**	1.70E-06
LRRC2	ENSG00000163827	**9.3**	5.78E-09	LCN15	ENSG00000177984	-5.0	4.83E-07
IGF1	ENSG00000017427	**8.9**	3.12E-17	SGSM1	ENSG00000167037	**-6.6**	3.08E-09
WNT5B	ENSG00000111186	**9.8**	1.00E-13	SEMA3D	ENSG00000153993	-5.3	2.34E-09
P2RY6	ENSG00000171631	**8.6**	5.24E-13	BMP3	ENSG00000152785	**-10.0**	2.15E-10
S1PR2	ENSG00000267534	**8.7**	4.01E-11	NFAM1	ENSG00000235568	-4.3	2.08E-11
CHAC1	ENSG00000128965	**8.4**	7.68E-15	LRRC70	ENSG00000186105	**-4.7**	1.97E-10
HTR4	ENSG00000164270	**10.9**	2.89E-11	KLF4	ENSG00000136826	**-7.3**	2.42E-14
EFEMP1	ENSG00000115380	**8.4**	3.39E-07	GALNTL6	ENSG00000174473	**-9.7**	1.15E-08
AC136616.2	ENSG00000277761	**10.3**	3.60E-10	ZNF703	ENSG00000183779	**-6.6**	2.82E-12
KCNA2	ENSG00000177301	**8.5**	9.31E-16	MDS2	ENSG00000197880	**-8.2**	3.22E-12
IGJ	ENSG00000132465	**8.4**	1.41E-16	ZDHHC19	ENSG00000163958	**-7.2**	1.38E-13
ZNF697	ENSG00000143067	8.9	2.12E-13	RGS7	ENSG00000182901	**-9.4**	7.39E-10
TSHR	ENSG00000165409	**8.9**	1.37E-12	GRASP	ENSG00000161835	**-7.3**	3.40E-13
HRASLS2	ENSG00000133328	8.6	3.30E-11	FOSB	ENSG00000125740	**-6.8**	4.97E-09
NLRP7	ENSG00000167634	8.8	2.92E-08	FOS	ENSG00000170345	**-8.0**	7.11E-10
IGSF3	ENSG00000143061	9.1	1.82E-11	KIAA1683	ENSG00000130518	**-5.2**	2.99E-16
TNFRSF9	ENSG00000049249	9.1	5.19E-13	IRS2	ENSG00000185950	-4.8	2.34E-15
NEIL3	ENSG00000109674	8.8	1.57E-13	DUSP1	ENSG00000120129	-7.7	1.42E-17
UNC13C	ENSG00000137766	9.2	7.30E-09	CYGB	ENSG00000161544	-4.6	3.86E-09
HIST1H3C	ENSG00000278272	9.0	1.09E-08	DOK7	ENSG00000175920	-4.9	1.05E-10
CDC6	ENSG00000094804	8.8	4.62E-19	GPA33	ENSG00000143167	-7.2	5.15E-12
HPGD	ENSG00000164120	8.5	9.58E-16	P2RY14	ENSG00000174944	**-8.1**	1.85E-16
HJURP	ENSG00000123485	9.1	8.54E-17	SLC11A1	ENSG00000018280	-4.7	4.56E-10
MCM10	ENSG00000065328	9.7	1.48E-17	DUSP8	ENSG00000184545	-5.0	3.28E-10
DEPDC1	ENSG00000024526	9.4	1.15E-16	ESPNL	ENSG00000144488	-5.8	9.36E-09
KIF18B	ENSG00000186185	8.7	1.89E-17	ZNF618	ENSG00000157657	-6.3	3.52E-14
ANLN	ENSG00000011426	8.6	1.90E-17	ARRDC4	ENSG00000140450	-5.3	1.65E-13
DLGAP5	ENSG00000126787	9.8	1.75E-18	VPREB3	ENSG00000128218	-5.5	1.33E-15
SPC25	ENSG00000152253	8.8	1.41E-16	CLEC4C	ENSG00000198178	-7.0	4.83E-11
CEP55	ENSG00000138180	9.4	3.36E-19	SYN3	ENSG00000185666	-6.3	2.74E-11
TICRR	ENSG00000140534	8.7	6.46E-18	IL13RA1	ENSG00000131724	-5.2	1.88E-15
KIF14	ENSG00000118193	9.0	2.18E-17	PRCD	ENSG00000214140	-5.5	1.06E-13
BIRC5	ENSG00000089685	8.6	1.07E-19	ZCCHC18	ENSG00000166707	-5.0	4.39E-14
KIF23	ENSG00000137807	8.5	1.13E-19	DNAJB13	ENSG00000187726	-4.8	7.36E-15
RRM2	ENSG00000171848	9.2	8.60E-17	ZBTB16	ENSG00000109906	-8.9	7.46E-16
MKI67	ENSG00000148773	8.9	4.61E-21	MEGF6	ENSG00000162591	-5.5	8.11E-16
CDC20	ENSG00000117399	8.5	3.22E-20	GPRASP1	ENSG00000198932	-5.0	1.75E-14

*p-value based on ANOVA-like QL F-test; Bold FC values indicate FC from D0 vs D10 IVPCs; Not bolded FC values indicate FC from D0 vs D4
